# Enhancing the freeze–thaw stability of maize starch via targeted mutation of both *Waxy1* and *Sugary2*

**DOI:** 10.1111/pbi.70140

**Published:** 2025-05-23

**Authors:** Mingzheng Ma, Peifeng Liu, Jinjie Zhu, Zhaoxu Gao, Xiantao Qi, Chuanxiao Xie, Changlin Liu

**Affiliations:** ^1^ State Key Laboratory of Crop Gene Resources and Breeding Institute of Crop Science, Chinese Academy of Agricultural Sciences Beijing China; ^2^ Hainan Yazhou Bay Seed Lab Sanya Hainan China

**Keywords:** Maize, CRISPR/Cas9, freeze–thaw stability, *Waxy1*, *Sugary2*

Frozen foods have emerged as staples in household shopping baskets. However, these foods often endure a series of temperature shifts during storage, transportation and freeze–thaw cycles, leading to quality degradation manifested by moisture loss, surface cracking and hardening. This deterioration could be alleviated by the addition of starches with robust freeze–thaw stability (Kowalski *et al*., 2017). Maize (*Zea mays* L.), a staple crop worldwide, consists of approximately 70% starch. It stands out as the predominant source for producing starches with robust freeze–thaw stability. The freeze–thaw stability of starch was mainly influenced by its structure. The *Waxy1* gene in maize encodes granule‐bound starch synthase, and its inactivation inhibits the amylose synthesis pathway, yielding starch with almost all amylopectin (Shure *et al*., 1983). The *Sugary2* gene encodes SSIIa in soluble starch synthase, and its mutation reduces the proportion of medium‐chain amylopectin in starch, increasing the proportion of short amylopectin branches (Zhang *et al*., 2004). In the present study, we found that the freeze–thaw stability of maize starch could be significantly improved via targeted mutation of both *Waxy1* and *Sugary2* using CRISPR/Cas9.

We constructed a vector that simultaneously expressed Cas9 and two sgRNAs, targeting *Waxy1* and *Sugary2*, respectively (Figure 1a,b). After the transformation of maize inbred line KN5585, a diversity of mutations was identified in the targeted regions of *Waxy1* and *Sugary2* in the seedlings of the T_0_ generation, as determined by targeted PCR amplification and sanger sequencing. Eight types of transgene‐free frameshift mutations were employed for further study (Figure 1c), including three types of mutations in *Sugary2* (S1, S2, S3), two types of mutations in *Waxy1* (W1, W2) and three types of double‐gene mutations (SW1, SW2, SW3). Compared with the functional Waxy1 and Sugary2 proteins, all frameshift mutations were predicted to produce proteins lacking the key catalytic domain. These results were validated by the significant reduction of granule‐bound starch synthase activity in the *Waxy1* mutants and soluble starch synthase activity in the *Sugary2* mutants (Figure S1).

**Figure 1:**
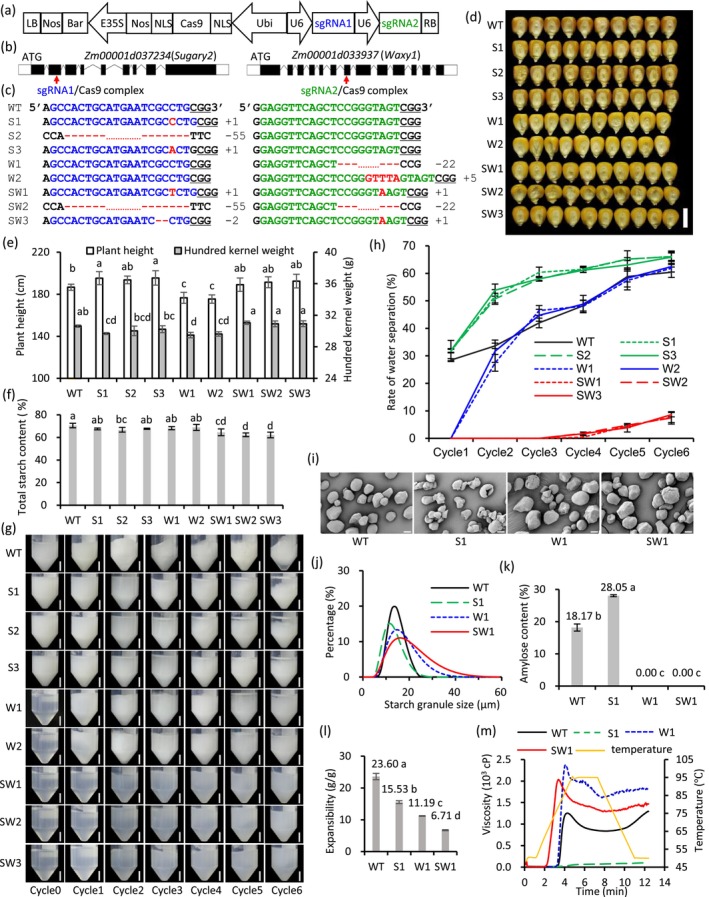
Improvement of the freeze–thaw stability of maize starch through precisely mutating both *Waxy1* and *Sugary2*. (a) The vector used to mutate both *Waxy1* and *Sugary2*, described by Li *et al*. (2017). (b) The structures of the two genes and the designed two target sites. (c) Genotypes of selected homozygous mutations. Kernels (d), plant height (e), hundred kernel weight (e), total starch content of kernels (f) of the WT and its mutations in T_3_ generation. (g) Six cycles of freeze–thaw of the starches. (h) The rate of water loss of the starches during six cycles of freeze–thaw. (i) Scanning electron micrographs of purified starch granules. Starch granule size (j), amylose content (k), starch expansibility (l) and starch viscosity (m) of the WT and its mutations. Scale bars in (d), (g) and (i) were 1 cm, 1 cm and 10 μm, respectively. The appended different letters in (e), (f), (k) and (l) signify statistically significant differences determined using Duncan's New Multiple Range Test at *P* = 0.05.

The agronomic traits of mutants were evaluated in the T_3_ generation. Changes in the appearance of grains, as well as plant phenotypes including plant height and ear height, were observed in lines with mutations in either *Waxy1* or *Sugary2* (Figure 1d,e; Table S1). These observations may be due to mutation effects, residual heterozygosity (5.56% in KN5585; Table S3) and/or environmental factors. For the plants of the double‐gene mutants, the plant height and hundred‐kernel weight were increased compared to the wild type (WT) KN5585, but the difference was not significant. No significant difference was detected among the mutants within the same genes with regard to the plant phenotypes and the appearance of grains (Figure 1d,e; Table S1). However, mutant kernels exhibited a reduction in total starch content compared to WT (Figure 1f; Table S1).

The freeze–thaw stability of starches was assessed (Kowalski *et al*., 2017). By examining the state diagrams of the starch gels after each freeze–thaw cycle (Figure 1g), it was evident that following cycle0 (post‐gelatinization), both the WT and the mutants of *Sugary2* exhibited opaque white starch solutions with relatively low viscosity and demonstrated retrogradation upon standing. Conversely, the mutants of *Waxy1* and the double‐gene mutants presented as translucent, highly viscous, and stable colloidal solutions. After the initial freeze–thaw cycle, a decrease in transparency was observed in the starch gels of the mutants of *Waxy1* and the double‐gene mutants. The WT exhibited an overall spongy state post the first cycle. The mutants of *Sugary2* displayed a flocculent sponge‐like structure after the second cycle. They were clearly discernible in the third cycle, along with evident water accumulation on the upper layer. As the freeze–thaw cycles progressed, the double‐gene mutants transitioned from a highly transparent colloidal solution to an opaque, whitish colloidal solution. They maintained no water loss in the first 3 cycles and only a minimal amount of water separation from the upper part of the colloidal solution in the sixth cycle, demonstrating exceptional water‐holding capacity.

The amount of water expelled via syneresis was quantified following each freeze–thaw cycle (Figure 1h) (Srichuwong *et al*., 2012). Consistent conclusions could be drawn from the graph. For the WT, the rate of water separation rapidly increased in the first 5 cycles and slightly incremented further in the sixth cycle compared to the fifth. Ultimately, the rate of water separation reached approximately 60%. For the mutants of *Sugary2*, the rate of water separation rapidly increased in the first 2 cycles, surpassing that of the WT. In subsequent cycles, the growth rate of the water separation rate decelerated, culminating in a final rate of approximately 66%. For the mutants of *Waxy1*, the rate of water separation was lower than that of the WT in the first 2 cycles but surpassed it in the third cycle, concurrent with the clear observation of sponge‐like structures. Subsequent changes mirrored those of the WT. For the double‐gene mutants, no water separation occurred in the first 3 cycles, and an extremely low water separation rate was detected at the end of the fourth cycle. Even in the sixth cycle, the water separation rate remained below 10%, indicating that the double‐gene mutants possessed an exceptionally strong water‐holding capacity not exhibited by the WT and the other two kinds of mutants.

The represent mutants (S1, W1, SW1) and the WT were further employed to characterize starch granule morphology, amylose content, viscosity and expansibility (Ma *et al*., 2024). Scanning electron microscopy (SEM) micrographs revealed that the WT starch granules exhibited irregular, polyhedral shapes with sharp edges (Figure 1i). SW1 featured most starch granules with irregular shapes and rough surfaces, along with some relatively large starch granules. Aligning with the SEM observation results, when compared to the WT, SW1 showed a significant increase in the proportion of large starch granules (Figure 1j). The amylose content was consistent with the previous results of the mutants of *Waxy1* and(or) *Suary2* (Shure *et al*., 1983; Zhang *et al*., 2004) (Figure 1k). By examining the starch expansibility (Figure 1l), it was evident that the expansibility of S1 and W1 was considerably lower than that of the WT. Furthermore, the expansibility of SW1 was also notably decreased when compared to both S1 and W1 (*P* < 0.05). Based on the viscosity measurement data (Figure 1m; Table S2), the SW1 starches exhibited significant increases (*P* < 0.05) in peak viscosity, minimum viscosity, breakdown value and final viscosity compared to the WT samples. In contrast, the S1 starches showed significant decreases (*P* < 0.05) in all viscosity parameters compared to the WT samples.

In summary, we demonstrated that the freeze–thaw stability of maize starch could be greatly enhanced via targeted mutation of both *Waxy1* and *Sugary2*. The starch from the double‐gene mutants maintained its stability even after multiple freeze–thaw cycles. This starch was useful to alleviate quality degradation of frozen foods during storage, transportation and freeze–thaw cycles. Importantly, no agronomic penalty was observed in the plants of the double‐gene mutants. Therefore, this study provides a basis for rapidly improving the freeze–thaw stability of excellent maize lines using targeted mutation strategies.

## Conflict of interest

A related patent had been submitted to the State Intellectual Property Office of China.

## Supporting information


**Figure S1** The granule‐bound starch synthase activity and soluble starch synthase activity of the wild type and its mutations. The appended different letters signify statistically significant differences at *P* = 0.05.

**Table S1** The agronomic traits of the wild type and its mutations.

**Table S2** The starch viscosity of the wild type and its mutations.

**Table S3** The residual heterozygosity, and the percentage of SNPs with different genotypes, of the wild type and its mutations.

## Data Availability

The data that supports the findings of this study are available in the supplementary material of this article.
