# Supplementary figures and images for: Enhancing the freeze–thaw stability of maize starch via targeted mutation of both Waxy1 and Sugary2
Source: Plant Biotechnol J. 2025 May 23;23(8):3254–6. doi: 10.1111/pbi.70140 (PMC12310825; doi:10.1111/pbi.70140)

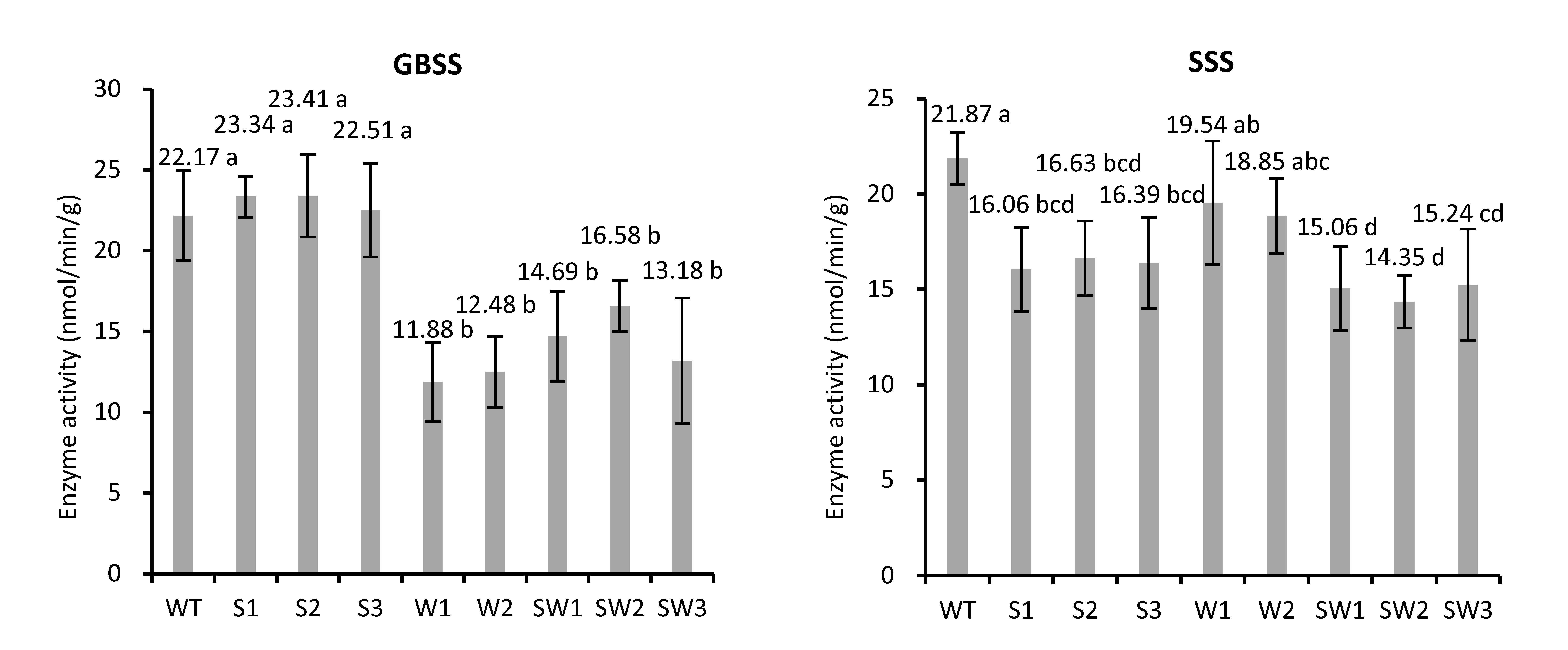

Supplement: Supplementary file 1 — Figure S1 The granule‐bound starch synthase activity and soluble starch synthase activity of the wild type and its mutations. The appended different letters signify statistically significant differences at P = 0.05. [file PBI-23-3254-s002.jpg]
